# sNebula, a network-based algorithm to predict binding between human leukocyte antigens and peptides

**DOI:** 10.1038/srep32115

**Published:** 2016-08-25

**Authors:** Heng Luo, Hao Ye, Hui Wen Ng, Sugunadevi Sakkiah, Donna L. Mendrick, Huixiao Hong

**Affiliations:** 1 National Center for Toxicological Research, U.S. Food and Drug Administration, 3900 NCTR Rd, Jefferson, AR 72079 USA; 2 University of Arkansas at Little Rock/University of Arkansas for Medical Sciences Bioinformatics Graduate Program, 2801 S University Ave, Little Rock, Arkansas, AR 72204 USA

## Abstract

Understanding the binding between human leukocyte antigens (HLAs) and peptides is important to understand the functioning of the immune system. Since it is time-consuming and costly to measure the binding between large numbers of HLAs and peptides, computational methods including machine learning models and network approaches have been developed to predict HLA-peptide binding. However, there are several limitations for the existing methods. We developed a network-based algorithm called sNebula to address these limitations. We curated qualitative Class I HLA-peptide binding data and demonstrated the prediction performance of sNebula on this dataset using leave-one-out cross-validation and five-fold cross-validations. This algorithm can predict not only peptides of different lengths and different types of HLAs, but also the peptides or HLAs that have no existing binding data. We believe sNebula is an effective method to predict HLA-peptide binding and thus improve our understanding of the immune system.

Human leukocyte antigens (HLAs) are the major histocompatibility complexes (MHCs) in humans. They are expressed on the surfaces of antigen presenting cells to recognize endogenous or foreign peptides for immunological reactions^1^,^2^. The genes that encode HLAs are a gene system located at the short arm of Chromosome 6. They are highly polymorphic across populations^3^,^4^,^5^. There are different classes of HLAs, including Class I, II and III, according to their genetic locations. Different classes of HLAs have divergent structures and functions. Both Class I and Class II HLAs have a long binding groove that can bind peptides and present them onto T-cell receptors^6^,^7^,^8^, while Class III HLAs are a part of the complement system to help with pathogen clearance^9^. Class I HLAs capture the endogenous peptides degraded from cytosolic proteins and present them to the T-cell receptors on the surface of CD8+ T-cells for cytotoxic responses, while the Class II HLAs present exogenous peptides from extracellular sources to the CD4+ T-cells to trigger acquired responses including antibody synthesis^10^,^11^. The binding between Class I/II HLAs and peptides is an important process for immune responses. Studying HLA-peptide binding will help us better understand the immune system and the mechanisms of autoimmune diseases and adverse drug reactions^12^,^13^ and will also provide important information needed in the development of vaccines and protein therapeutics^14^,^15^.

Since HLA-peptide binding is important for immune-related applications, experimental binding assays were developed to test *in vitro* binding affinities between HLAs and peptides and the data were collected in databases such as AntiJen^16^, IEDB^17^, MHCBN^18^ and SYFPEITHI^19^. The IMGT/HLA database recorded more than 13,000 HLA alleles by August 2015^20^. Since it is time-consuming and costly to experimentally test the binding between large numbers of HLAs and peptides, computational methods have been developed to predict HLA-peptide binding^21^. The current widely used methods are machine learning methods; however, several challenges limit their applicability. First, many machine learning methods can only predict a limited number of HLAs or peptides with a specific length. Second, an HLA-specific model would be unreliable if the training samples were not large enough^21^. Therefore, we developed the neighbor-edges based and unbiased leverage algorithm (Nebula) based on network analysis to overcome the limitations of machine learning methods^22^,^23^. We successfully applied Nebula to predict HLA-peptide binding and found that it delivered a reasonable performance. However, Nebula is not applicable to predict the binding between a peptide and an HLA if no experimental data are available between the peptide and other HLAs or no binding assay has been developed for the HLA. Thus, Nebula is not able to predict binding for unstudied peptides and HLAs, limiting its application. Nebula is an algorithm purely based on the topology of a network; alternatively, the network is treated as a colorless graph where the nodes are not differentiated (colorless). Actually, the nodes (HLAs and peptides) in the bipartite network of HLA-peptide could be differentiated in many ways. Thus, appropriate consideration of node difference in a prediction algorithm is expected to improve its performance. In this study, we developed a new network-based prediction algorithm called similar neighbor-edges based and unbiased leverage algorithm (sNebula) by presenting the bipartite network of HLA-peptide binding data in a color graph. By introducing color to the network as additional information, sNebula can predict binding activity for peptides and HLAs that are not included the training network, overcoming the limitation of Nebula. We used the qualitative binding data between Class I HLAs and peptides as an example. We demonstrated that sNebula is a reliable algorithm for prediction of HLA-peptide binding and can be applied to HLAs or peptides with or without experimental binding data.

## Results

### Data curation

We curated 43,935 peptides, 135 Class I HLAs and 141,224 qualitative HLA-peptide binding data from the four databases. The binding data are given in Supplementary Table S1. Among the 43,935 distinct peptides, the peptide length varies from 6 to 30. Most of the peptides are 9-mers (65%) and 10-mers (25%), which is consistent with the experimental discovery of Class I HLA-binding peptides^24^. The distribution of peptide lengths is summarized in Supplementary Table S2. The 135 HLAs include 49 *HLA-A* alleles, 75 *HLA-B* alleles, 9 *HLA-C* alleles and 2 *HLA-E* alleles. The HLA alleles and their pseudo-sequences are listed in Supplementary Table S3. Among the 141,224 HLA-peptide binding data, 47% are bindings and 53% are non-bindings.

### Leave-one-out (LOO) cross-validation

**Figure 1:**
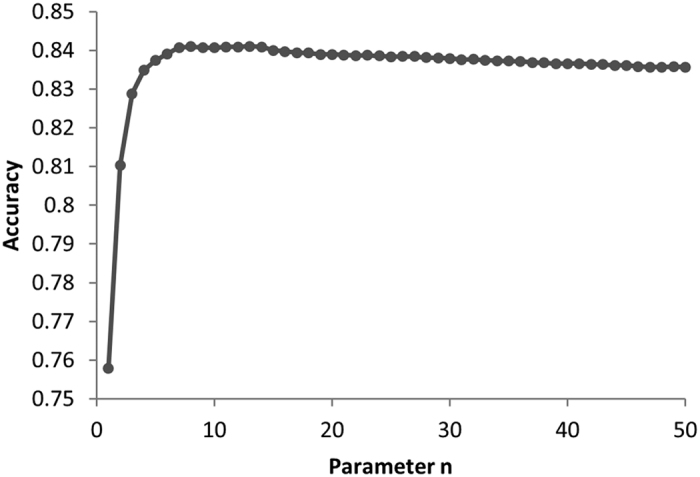
Determination of parameter *n* using LOO cross-validations for sNebula. The y-axis is the prediction accuracy and the x-axis indicates *n*.

Parameter *n* indicates the maximum of neighbors from the peptide and HLA that are used for sNebula to make a prediction of the binding between the HLA and the peptide. Fifty leave-one-out (LOO) cross-validations were conducted on the HLA-peptide binding data using parameter *n* = 1 to 50. The prediction accuracy values yielded from the 50 LOO cross-validations are shown in Fig. 1. When *n* = 13, the accuracy reached the maximal value 0.841, and the corresponding sensitivity, specificity and area under the receiver operating characteristic curve (AUC) values were 0.818, 0.862 and 0.841, respectively. As *n* increases after this point, the accuracy of the model gradually dropped. Thus we used *n* = 13 for LOO cross-validations.

### Five-fold cross-validations

**Figure 2:**
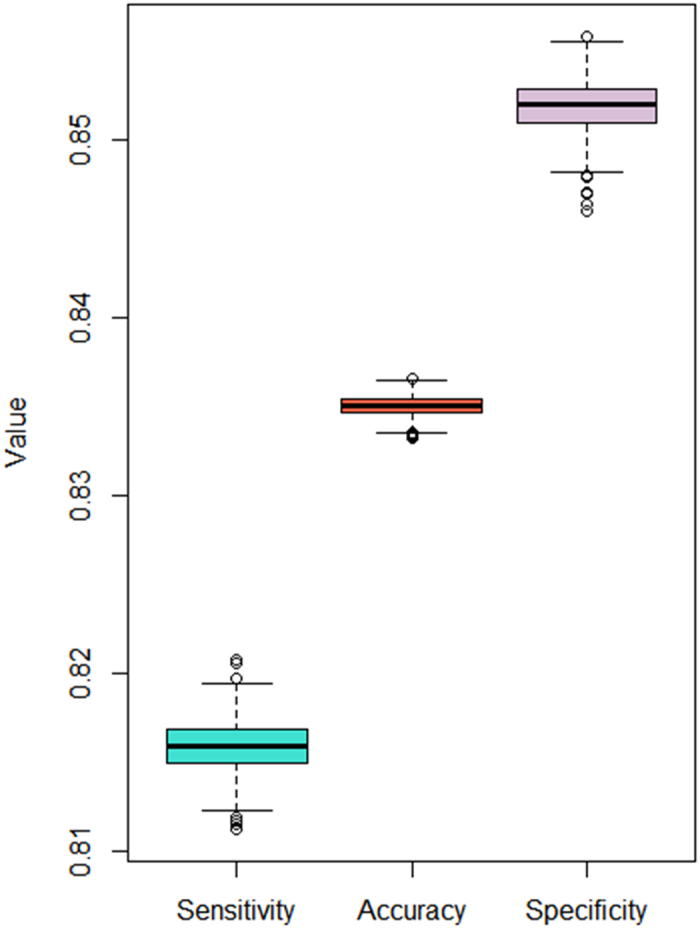
The distributions of sensitivity, specificity and accuracy seen in the 1,000 iterations of five-fold cross-validations.

One thousand iterations of five-fold cross-validations were conducted on the HLA-peptide binding data using sNebula. The prediction values in each of the five-fold cross-validations were compared with the experimental values and a set of accuracy, sensitivity and specificity values was calculated. The distributions of 1,000 values of these performance metrics are shown in Fig. 2. The average sensitivity, specificity and accuracy values are 0.816, 0.852 and 0.835, respectively, with the same standard deviation of 0.001.

### Confidence analysis

**Figure 3:**
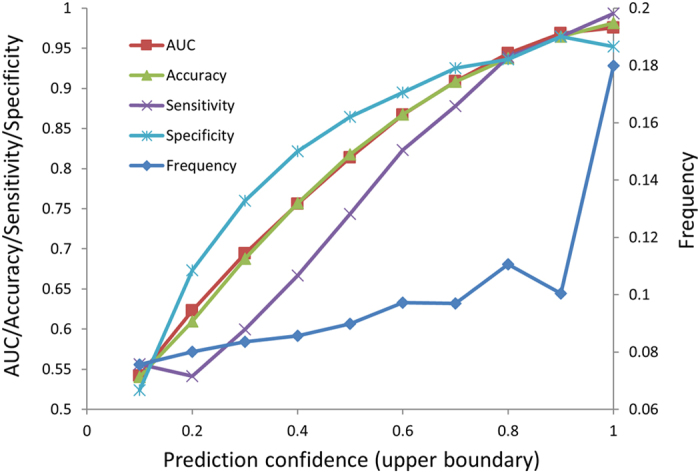
The relationships between prediction confidence and sNebula performance (AUC, accuracy, sensitivity, specificity) and prediction frequency. The confidence values ranging from 0 to 1 are grouped into 10 bins. The X-axis represents the upper boundary of each confidence bin. The left Y-axis indicates AUC, accuracy, sensitivity and specificity. The right Y-axis gives prediction frequency.

The sNebula predictions are continuous values that not only indicate binding status of binder/non-binder but also represent the prediction confidence levels. The confidence levels of sNebula predictions from the 1,000 iterations of five-fold cross-validations were calculated and used to place the predictions into 10 groups by confidence. The performance of sNebula was assessed for each of the 10 groups of predictions. The performance in terms of accuracy, sensitivity, specificity and AUC at different confidence levels were plotted in Fig. 3. As the confidence increased, the AUC, accuracy, sensitivity and specificity (indicated by the left y-axis) improved, and the predictions in number (indicated by the right y-axis) also increased. The confidence analysis results revealed that the higher the prediction confidence level the better the prediction performance of sNebula. Moreover, most predictions from sNebula were at high confidence.

### Benchmark

The IEDB website contains the performance comparison of various prediction methods for HLA-peptide binding (http://tools.iedb.org/auto_bench/mhci/weekly/). We used NetMHCpan^25^,^26^,^27^ to compare with sNebula and Nebula. The performance comparison is shown in Table 1. Different methods had different performance depending on the dataset and the HLA. While NetMHCpan performed well on some datasets such as B^*^07:02, B*15:02 and B*27:04 in terms of AUC values, sNebula had better results on datasets such as C*03:04 and A*02:06. As a comparison, Nebula also had high AUC values on some datasets such as A*68:02 and B*38:01. However, because Nebula could not make predictions on HLAs and peptides that are not included in the training network, the results of Nebula were not complete for some datasets as B*27:04 and B*27:05.

## Discussion

The human HLA loci are in a genomic region that is among the most polymorphic. The HLA loci have retained much variation^28^,^29^,^30^. Thousands of HLA alleles have been discovered, including approximately 9000 alleles of Class I HLAs^20^,^31^. The proteins encoded by HLAs are used by the immune system to recognize invaders such as foreign pathogens. However, the proteins themselves are not able to display biological functions. The binding groove of HLA proteins holds a peptide that can exhibit functions of HLAs such as social recognition skills^32^. It follows that knowledge of HLA-peptide binding plays a key role in understanding related biomedical questions such as autoimmune diseases and HLA-mediated adverse drug reactions^33^,^34^. Many *in vitro* experiments have been designed to assay HLA-peptide binding^35^. However, due to the huge number of possible binding interactions between thousands of HLAs and millions of peptides, it is difficult, if not impossible, to comprehensively ascertain the binding interactions between HLAs and peptides. Thus, computational methods can play a crucial role in the study of HLA-peptide binding. Though some computational approaches have been proposed for prediction of HLA-peptide binding^21^,^36^, the practicability is limited since many methods do not support HLAs with few binding peptides or peptides that are diverse in length. Some recently developed methods such as NetMHCpan^25^,^26^,^27^, NetMHC^37^ and kernel functions^25^,^38^ can predict for peptides with different lengths; however, extra processes^39^,^40^ are usually required to identify core binding sequences within the peptides so that they can be converted to a fixed length. Though such extra processes may not necessarily reduce performance of such methods, algorithms that can overcome some restrictions of the current computational methods and handle peptides with different lengths are expected to have wider applications. Using sNebula, one can generate a comprehensive atlas of binding interactions between HLAs and peptides. Based on bipartite network analysis, sNebula has no limitations on the number of HLA molecules used or the size of the peptides utilized for training and, thus, provides a promising solution for the construction of a comprehensive atlas of HLA-peptide binding. However, different from machine learning-based methods such as NetMHCpan^25^,^26^,^27^, sNebula is unable to directly predict HLA-peptide binding if neither the peptide nor the HLA exists in the training network.

The results of this current study suggest that sNebula can accurately predict the binding activity between HLAs and peptides, even though this is a very sparse dataset. The algorithm is useful because it can not only make predictions for untested peptides and HLAs given sequence information, but also can make more accurate predictions with a higher confidence. In addition, it does not set any limitation on the peptide length or the number of HLA alleles. With all these advantages, sNebula can help us study the binding between HLAs and peptides and improve our understanding of the immune system. Like HLA-peptide binding data, a lot of big data are diverse and incomplete^41^ such as gene expression data^42^,^43^, drug-target binding^44^ and clinical information^45^. Methods have been developed to impute the missing values for analysis including unsupervised and supervised classifications^43^,^46^,^47^. However, unlike the classification models, sNebula can deal with sparse or incomplete data without requiring the process of missing data imputation. It also accepts the diversity and flexibility of data so an assured length of features is not required. With the arising of big data era and increasing needs of big data analysis, we believe sNebula is one of the possible solutions to deal with large, diverse and incomplete data for predictions and novel discoveries.

Future applications of sNebula remain to be explored. In this study, sequences were utilized to calculate the similarity between nodes. It is possible to use sNebula in the development of similar algorithms for other applications. For example, it is possible to utilize the 2D structural fingerprints of drugs to replace sequences of peptides for similarity calculation and, thus, modify sNebula to predict drug-HLA binding or even drug-target binding that may underlie some observed genetic links to adverse drug events. Network-based inference (NBI) is a powerful network approach that can integrate a variety of data sources for a wide spectrum of applications such as drug-target predictions^48^,^49^, drug safety assessment^50^,^51^,^52^, driver mutations prioritization in cancer genomics^53^, RNA network prediction^54^ and xenobiotics gene and disease prediction^55^. Cheng *et al.* utilized Node Weighted Network-based Inference (NWNBI) to predict drug-target interactions using a node-weighted network and observed a better performance than the unweighted NBI^49^,^56^. They calculated the drug similarity by 2D fingerprints and target similarity by sequences. However, their method uses all the neighbors for prediction instead of selecting the top similar ones. It is possible to improve the prediction performance by selecting top similar neighbors using sNebula. Another possible application for sNebula is to predict drug-disease association for drug repurposing. Gottlieb *et al.* collected a network of drug-disease associations as well as information of drug-drug similarities and disease-disease similarities to predict novel drug-disease associations using logistic regression^57^. The machine learning method is useful; however, there are some challenges such as problems to deal with a flexible length of features^21^ or missing data^45^. Since sNebula is based on similarity and does not require the completeness or an assured length of features, it is possible to extend sNebula to predict drug-disease associations while overcoming those problems.

Another potential application of sNebula is to develop new therapeutics such as tumor immunotherapy. The neoantigens are peptides in the human body that are not encoded by the normal human genome. In tumors, they are generated by the tumor-specific DNA alternations^58^. When the gene expression data for patients are available, predicting HLA-peptide binding may help to identify or filter patient-specific neoantigens, which are a major factor for clinical immunotherapy development^58^,^59^. As more HLA-peptide binding data and patient-specific RNA sequencing data are becoming available, we believe sNebula can potentially help with neoantigen identification and the development of immunotherapies.

In addition to predictions values, sNebula also provides the confidence values. Confidence values are estimations about how likely the result is true; therefore, users can differentiate the results using confidence values and select the most confident predictions for validation. A good method not only makes more predictions in number, but also predicts with higher accuracy at higher confidence. From the confidence analysis result of sNebula, we saw sNebula predicted more and performed better with higher confidence. We believe the confidence values are useful information that can potentially help with the selection of prediction results for experimental validation in applications such as HLA-peptide binding, drug-target binding and drug-disease associations.

## Conclusion

We developed a network-based prediction algorithm, sNebula, to predict the binding potential between HLAs and peptides. We found this algorithm exhibited a good performance in both the LOO cross-validation and five-fold cross-validations using the experimental HLA-peptide binding data curated from major databases. The confidence analysis indicated its ability to make predictions with more accuracy when the confidence level is higher. This algorithm not only overcomes the limitations of the current machine learning methods on the number of HLAs and lengths of peptides, but also makes it possible to predict HLA-peptide binding for new peptides or HLAs. It could be expected that sNebula can help with the construction of a comprehensive atlas of HLA-peptide binding that, in turn, facilitates better understanding of the immune system.

## Methods

### Study design

**Figure 4:**
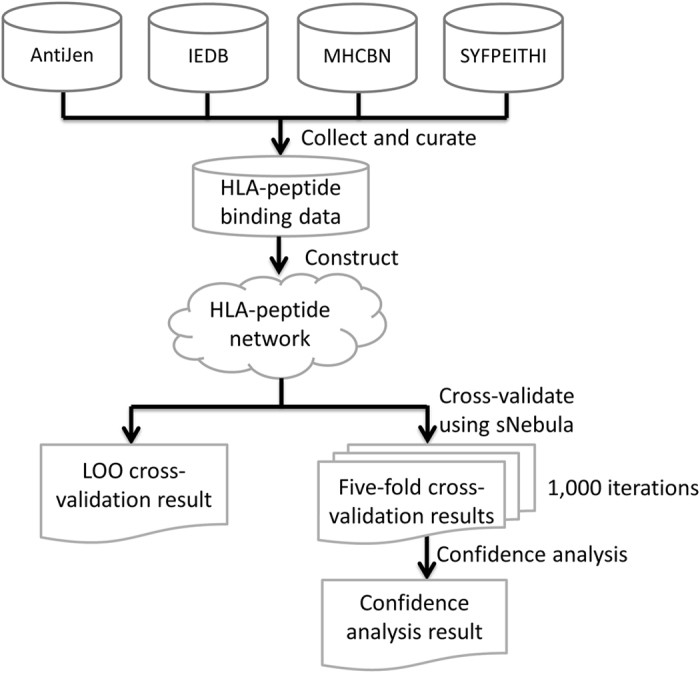
Study workflow. The qualitative Class I HLA-peptide binding data were curated from four major databases (AntiJen, IEDB, MHCBN and SYFPEITHI). A HLA-peptide binding data network was constructed based on the curated data. To assess the performance of sNebula, LOO cross-validation and 1,000 iterations of five-fold cross-validations were executed. Based on the results of five-fold cross-validations, confidence analysis was conducted to evaluate the relationship between the confidence levels and the prediction performance of sNebula.

The workflow of the study is shown in Fig. 4. Qualitative Class I HLA-peptide binding data were collected and curated from four databases: AntiJen^16^, IEDB^17^, MHCBN^18^ and SYFPEITHI^19^. A bipartite network of HLA-peptide binding data was then constructed. The binding data network was used to assess the performance of sNebula using leave-one-out (LOO) cross-validation and 1,000 iterations of five-fold cross-validations. The prediction confidence analysis was conducted based on the results of five-fold cross-validations.

### Data curation

The experimental Class I HLA-peptide binding data were collected from four databases (AntiJen^16^, IEDB^17^, MHCBN^18^ and SYFPEITHI^19^) as described in our previous study^22^. The databases provide qualitative binding categories (binding versus non-binding) for each HLA-peptide pair. We merged the four databases and recorded only one qualitative datum for each of HLA-peptide pairs using the majority voting strategy^22^. Since sNebula is based on a color graph of the network in which the nodes (HLAs and peptides) are colored using their amino acid sequences, we downloaded HLA sequences from the IMGT/HLA database^20^ and removed the HLAs that do not have an affirmative protein sequence such as HLA serotypes and allele groups and their binding peptides. The curated HLA-peptide binding data were used to construct a bipartite binding network, where HLAs and peptides are nodes, and the binding data between them, either binding or non-binding, are edges.

### sNebula

To predict the binding between peptide *p*_*x*_ and HLA *h*_*y*_, sNebula first identifies the peptides that are connected to HLA *h*_*y*_, *p*_*i*_ (*i* = 1, 2, …). The similarity between peptide *p*_*x*_ and each of the connected peptides of *h*_*y*_, *p*_*i*_, is calculated based on their sequences. To calculate the similarity between peptide *p*_*x*_ and peptide *p*_*i*_, sNebula first aligns the sequences of the two peptides without opening gaps and then calculates the similarity score *sp*_*x,i*_ using equations (1–2).


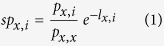



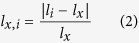


In equation (2), *l*_*x*_ and *l*_*i*_ are the lengths of peptides *p*_*x*_ and *p*_*i*_, respectively. In equation (1), *p*_*x,i*_ is the sequence similarity between peptides *p*_*x*_ and *p*_*i*_ and is calculated using BLOSUM50 matrix^60^, and *p*_*x,x*_ is the sequence similarity between peptide *p*_*x*_ and itself. BLOSUM50 matrix was used in this study because it has been used in many other methods for predicting HLA-peptide binding^26^,^39^,^61^,^62^. For each of possible alignments between peptide *p*_*x*_ and peptide *p*_*i*_, a sequence similarity value, *p*_*x,i*_, is calculated. The alignment with the largest sequence similarity value is then selected and its similarity, *p*_*x,i*_, is used for the calculation of equation (1).

In the same way, sNebula recognizes the HLAs that are connected to peptide *p*_*x*_, *h*_*j*_ (*j* = 1, 2, …). The similarity between HLA *h*_*y*_ and each of the connected HLAs of peptide *p*_*x*_, *h*_*j*_, is then calculated. For the HLA similarity calculation, instead of using HLA full sequences, sNebula utilizes the 48 unique residues on HLAs that closely interact with peptides which were identified by Chelvanayagam^63^ as HLA pseudo-sequences. The pseudo-sequence similarity score *sh*_*y,j*_ between HLA *h*_*y*_ and HLA *h*_*j*_ is calculated using equation (3).


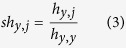


In equation (3), *h*_*y,j*_ is the pseudo-sequence similarity between HLA *h*_*y*_ and HLA *h*_*j*_ and is calculated using BLOSUM50 matrix, and *h*_*y,y*_ is the pseudo-sequence similarity between HLA *h*_*y*_ and itself. It is noted that both *sp*_*x,i*_ and *sh*_*y,j*_ are directional; thus, *sp*_*x,I*_ (*sh*_*y,j*_) is not necessarily equal to *sp*_*j,x*_ (*sh*_*j,y*_).

After the similarity scores for the nodes (HLAs and peptides) of the neighbor edges are calculated, sNebula ranks the peptides and HLAs using their similarity scores *sp*_*x,i*_ and *sh*_*y,j*_ to select *n* top ranked peptides and HLAs for *p*_*x*_ and *h*_*y*_, respectively, for the calculation of a continuous value *p*_*x,y*_ using equation (4) as the prediction of binding between *p*_*x*_ and *h*_*y*_. Here *n* is a parameter to be determined. The *n* with the best prediction of accuracy of LOO cross-validation based on the network of HLA-peptide binding data is used.


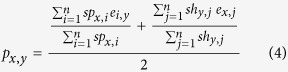


In equation (4), *e*_*i,y*_ is the binding edge weight between peptide *i* and HLA *y* in the HLA-peptide binding data network given by equation (5).


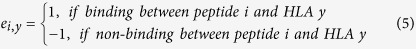


As an unbiased approach, sNebula considers the contribution from the peptides *p*_*x*_ and HLAs *h*_*y*_ of the neighbor edges equally. When peptide *p*_*x*_ do not contain neighbor edges, sNebula predicts the binding between peptide *p*_*x*_ and HLA *h*_*y*_ using equation (6).


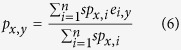


Therefore, if a peptide has no experiment data against any HLA in the training network, sNebula is still able to predict its binding towards the HLAs based on its sequence and the topological feature of the training network using equation (6). In the same way, if HLA *h*_*y*_ do not have neighbor edges, sNebula predicts the binding between peptide *p*_*x*_ and HLA *h*_*y*_ using equation (7).


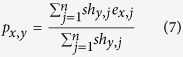


When the number of available connecting nodes (HLAs or peptides) for peptide *p*_*x*_ or HLA *h*_*y*_ is less than *n*, all of the nodes are used. When multiple connecting nodes for peptide *p*_*x*_ or HLA *h*_*y*_ have the same similarity score to be selected as the top *n* similar nodes, the average of their binding edge weights is used in equations (4), (6) and (7).

The binding prediction *p*_*x,y*_ is a continuous value between −1 and 1 and is converted into a categorical prediction value *c*_*x,y*_ using equation (8).


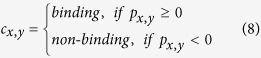


The goodness of the prediction *c*_*x,y*_ is assessed using a metric term as prediction confidence *conf*_*x,y*_ that is defined in equation (9).


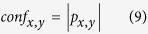


### LOO cross-validation

In the LOO cross-validation, each of the HLA-peptide binding data was taken out and the remaining HLA-peptide binding data were used to construct the HLA-peptide binding network for prediction of the binding value for the taken-out HLA-peptide pair using sNebula. This process was repeated until every HLA-peptide binding data were used as a test sample. The predicted values were then compared with the actual experimental binding data and sensitivity, specificity, accuracy and area under receiver operating characteristic curve (AUC) were calculated to evaluate the performance of sNebula. To determine the parameter *n*, we repeated the LOO cross-validation for *n* = 1, 2, 3 … 50. The *n* value with the highest prediction accuracy of LOO cross-validation was selected to be the parameter for sNebula.

### Five-fold cross-validations

We conducted 1,000 iterations of five-fold cross-validations on the HLA-peptide binding data to obtain a statistically robust estimation of sNebula performance. In a five-fold cross-validation, the HLA-peptide binding data were randomly divided into five parts as equal as possible. One part of HLA-peptide binding data was taken out to be used as test samples and the remaining four parts of HLA-peptide binding data were used as the training samples to construct a bipartite network. LOO cross-validations were conducted on training samples to determine the parameter *n* in sNebula. The network constructed from the training samples and the determined parameter *n* was used by sNebula to predict HLA-peptide binding of the test samples. This process was repeated five times so that each of the five parts of the HLA-peptide binding data was used once and only once as test samples. The categorical prediction results from all the five folds of test samples were compared to the actual experimental HLA-peptide binding data to calculate the sensitivity, specificity and accuracy to estimate the performance of sNebula.

### Prediction confidence analysis

Prediction confidence has been proposed as one of the metrics to measure performance of predictive models developed in the FDA’s endocrine disruptors knowledge based project^64^,^65^,^66^,^67^,^68^,^69^,^70^,^71^ using variety of machine learning methods such as decision tree^72^, Decision Forest models^73^,^74^,^75^,^76^,^77^,^78^ based on molecular descriptors^79^ that are calculated using the algorithm developed for the expert systems of structure elucidation^80^,^81^,^82^,^83^,^84^,^85^, support vector machine^86^,^87^ and principal component analysis based algorithm^88^,^89^. The continuous value output from sNebula for prediction of binding between an HLA and a peptide is the measure of likelihood of the peptide is a binder or non-binder of the HLA and indicates the confidence for the prediction. A good prediction method should not only show an overall high prediction accuracy but also is expected to 1) predict most unknown samples with a high confidence and 2) show a higher accuracy for the predictions with a higher confidence than the predictions with a lower confidence. We examined the relationship between prediction confidence and accuracy using all predictions from the 1,000 iterations of five-fold cross-validations. The prediction confidence was calculated using equation (9) for every prediction. The predictions were then placed into 10 groups with even confidence bins according to their confidence values. For each of the 10 groups of predictions, we calculated the sensitivity, specificity, accuracy and AUC by comparing the predictions with the actual experimental HLA-peptide binding data. At last, the performance of sNebula at difference confidence levels was analyzed.

### Benchmark

IEDB has an automatic server benchmark page (http://tools.iedb.org/auto_bench/mhci/weekly/) that evaluates different prediction methods for HLA-peptide binding based on new data submitted in the past three months or last week. We used the latest three datasets of 3-month period (2016-05-03, 2016-02-19 and 2015-08-07) as an example to compare sNebula with existing methods as well as its predecessor, Nebula. The parameter *n* was set to 13 for sNebula to make predictions. Two performance metrics, Spearman’s Rank Correlation Coefficient (SRCC) and AUC, were calculated between the predicted values and experimental values using R.

## Additional Information

**How to cite this article**: Luo, H. *et al.* sNebula, a network-based algorithm to predict binding between human leukocyte antigens and peptides. *Sci. Rep.* **6**, 32115; doi: 10.1038/srep32115 (2016).

## Supplementary Material

Supplementary Information

## Figures and Tables

**Table 1:** Performance comparison of NetMHCpan, sNebula and Nebula on IEDB benchmark datasets.

Dataset	IEDB reference	HLA	Peptide length	Peptide count	Measurement	NetMHCpan		sNebula		Nebula	
						SRCC	AUC	SRCC	AUC	SRCC	AUC
2016-05-03/2016-02-19	1029957	B*38:01	9	28	ic50	0.766	0.963	0.171	0.531	0.400	1.000
	1029824	A*02:01	9	77	binary	0.071	0.546	0.060	0.539	—	—
2015-08-07	1027131	B*15:02	9	14	binary	0.713	1.000	0.693	0.939	−0.707	0.000
	1029125	B*27:04	9	21	binary	0.717	0.939	0.133	0.582	—	—
	1029125	B*27:05	9	21	binary	0.751	0.959	0.752	0.959	—	—
	1029125	B*27:06	9	21	binary	0.421	0.750	0.421	0.750	0.500	0.750
	1029061	B*57:01	9	26	ic50	0.612	0.943	0.169	0.575	0.000	0.250
	315209	C*03:04	9	14	t1/2	0.781	0.911	0.113	0.923	—	—
	1028928	A*02:01	9	13	binary	0.570	0.955	0.539	0.909	—	—
	1028928	B*07:02	9	12	binary	0.648	1.000	0.522	0.900	—	—
	315174	B*27:03	9	11	binary	0.657	0.893	0.179	0.607	0.436	0.750
	1028790	A*02:01	9	55	ic50	0.615	0.574	0.505	0.778	0.478	0.856
	1028790	A*02:01	10	35	ic50	0.407	0.677	0.432	0.704	0.528	0.725
	1028790	A*02:02	9	55	ic50	0.582	0.713	0.372	0.680	0.427	0.668
	1028790	A*02:03	9	55	ic50	0.539	0.696	0.477	0.629	0.450	0.757
	1028790	A*02:03	10	35	ic50	0.208	0.750	0.419	0.697	0.308	0.691
	1028790	A*02:06	9	55	ic50	0.630	0.770	0.510	0.848	0.537	0.795
	1028790	A*02:06	10	35	ic50	0.572	0.768	0.525	0.680	0.502	0.700
	1028790	A*68:02	9	55	ic50	0.534	0.806	0.482	0.713	0.545	0.889
	1028790	A*68:02	10	35	ic50	0.272	0.620	0.591	0.813	0.533	1.000

The datasets along with the performance metrics of NetMHCpan were harvested from IEDB automatic server benchmark page (http://tools.iedb.org/auto_bench/mhci/weekly/). SRCC stands for Spearman’s Rank Correlation Coefficient and AUC stands for area under the receiver operating characteristic curve. The “–” mark means not applicable.
